# Surveillance of antimicrobial consumption in animal production sectors of low- and middle-income countries: Optimizing use and addressing antimicrobial resistance

**DOI:** 10.1371/journal.pmed.1002521

**Published:** 2018-03-01

**Authors:** Daniel Schar, Angkana Sommanustweechai, Ramanan Laxminarayan, Viroj Tangcharoensathien

**Affiliations:** 1 U.S. Agency for International Development, Bangkok, Thailand; 2 International Health Policy Program, Ministry of Public Health, Bangkok, Thailand; 3 London School of Hygiene and Tropical Medicine, London, United Kingdom; 4 Center for Disease Dynamics, Economics & Policy, Washington DC, United States of America; 5 Princeton Environmental Institute, Princeton University, Princeton, New Jersey, United States of America

## Abstract

In a policy forum, Daniel Schar and colleagues discuss the need for surveillance of antimicrobial consumption in animals in low- and middle-income countries and propose the establishment of antimicrobial consumption monitoring systems.

**Summary points**

- Antimicrobial use in low- and middle-income country (LMIC) food animal production sectors is accelerating, commensurate with expanded intensive production to meet rapidly increasing demand for animal-source nutrition.
- However, antimicrobial consumption in animal production contexts of LMICs remains largely undocumented, limiting the ability to establish and monitor progress toward achieving consumption targets.
- We propose the establishment of antimicrobial consumption monitoring systems in a phased manner that will be responsive and adaptive to LMIC contexts, while directing a path toward incremental enhancement of monitoring structures.
- This phased approach enables implementation of systems yielding standardized, globally comparable antimicrobial consumption data, which could inform policies to optimize antimicrobial usage in food animal production.
- The approach should be complemented by efforts to strengthen animal production systems, eliminate medically important antimicrobial growth promoters, and reduce reliance upon prophylactic antimicrobial use.

Global antimicrobial consumption in terrestrial and aquatic food animal production is accelerating, associated with expanded production to meet increasing demand for animal-source nutrition [1]. In South Asia, for example, demand for poultry between 2000 and 2030 is expected to increase by 725% [2]. Overall, growth in demand for animal-source nutrition through 2030 is anticipated to be higher in low- and middle-income countries (LMICs) as compared with high-income countries [2].

Efforts to meet rising demand for animal-source nutrition in LMICs are driving a shift in animal production from small holder, mixed crop, and livestock operations to increasingly intensive, large-scale, and specialized commercialization [1,3]. Intensive production systems have historically employed nontherapeutic antimicrobial use, particularly during transitionary stages [1]. Nontherapeutic antimicrobial use includes both mass administration for prevention and control of disease and antimicrobial growth promoter (AGP) use. AGPs are antimicrobials administered at subtherapeutic doses, are intended to enhance growth and production efficiencies, and are generally delivered in feed or water for extended duration. Although AGPs are not authorized in many countries, LMIC regulatory structures are often insufficient to monitor and enforce AGP bans.

Quantitative volumes of AGP usage from LMICs are not available. However, studies from LMICs indicate substantial nontherapeutic use. In the Mekong Delta of Vietnam, 84% of poultry farms surveyed indicated that antimicrobials were used for prophylactic rather than therapeutic purposes, and nearly a third of overall usage involved antimicrobial classes on the WHO list of highest priority, critically important antimicrobials for human medicine [4]. As a consequence of this changing landscape of animal production, without appropriate policy to optimize use, global antimicrobial consumption in food-producing animals, estimated at 131,109 tons in 2013, is projected to increase over 50% by 2030 to 200,235 tons [5]. In the BRICS countries—Brazil, Russia, India, China, and South Africa—antimicrobial consumption in food animals is projected to double [1].

The discovery and commercialization of antimicrobials stands as a defining achievement of 20th century medicine. As usage patterns drive rising rates of antimicrobial resistance (AMR)-linked morbidity and mortality [6]—compounded by a formidable cross-sectoral economic burden [7]—the global community faces an unprecedented challenge of preserving—and maintaining in perpetuity—antimicrobial efficacy.

Antimicrobial usage (AMU) in humans and animals exerts selection pressure potentiating AMR [1,5–8]; policy goals aim to reduce antimicrobial use where effective alternatives are available. A standardized framework guiding collection of incrementally detailed, internationally comparable antimicrobial consumption data from animal production could accelerate progress toward optimizing global antimicrobial use (Box 1). Yet, no such standardized framework relevant to LMIC animal production contexts exists [9,10], challenging the characterization of usage and limiting government policies aimed at optimizing and monitoring AMU in animal production sectors.

**Box 1. Benefits expected from establishing a standardized framework for antimicrobial consumption in animal production**

1. Monitor consumption trends and benchmarking for optimization of antimicrobial consumption.
  - Facilitates identification of trends and usage profiles,
  - Permits establishment of time-bound consumption targets,
  - Monitors progress toward achieving targets,
  - Guides policy and targeted interventions optimizing antimicrobial use.
2. Comparison of antimicrobial consumption data across countries, species, farms, and with human consumption.
  - Comparison of consumption data ultimately disaggregated by species and comparable across human and animal sectors,
  - Antimicrobial consumption data become globally comparable,
  - Contributes to international benchmarking,
  - Creates a globally standardized monitoring architecture, upon which a future international agreement capping consumption could be developed.
3. Builds a platform for studying the association between antimicrobial consumption and AMR surveillance data.

## The challenge of establishing surveillance of antimicrobial consumption in animal production

The measurement of AMU in human health and animal health and production settings is a central goal of the Global Action Plan on Antimicrobial Resistance [11] and the complementary plans and strategies developed by the Food and Agriculture Organization of the United Nations (FAO) and World Organization for Animal Health (OIE) [12,13].

The ecosystem in which veterinary antimicrobials are produced, distributed, and utilized in LMICs is complex and variable. A majority of LMICs currently lack structures capable of quantifying consumption at sufficient resolution to provide usage data by antimicrobial class, animal species, production context, purpose of usage, and route of administration, which are necessary to facilitate effective interventions to optimize use [14]. Of the 74 LMIC OIE member states, 54 (73%) reported AMU to the OIE in 2015, while 20 (27%) provided no reporting at all. Of the 54 member states reporting, 30 (55.6%) were able to provide some quantitative usage data but were largely unable to report that data disaggregated by the aforementioned parameters [14].

Patterns of antimicrobial use differ widely across animal production value chains, as evidenced by macrolide, fluoroquinolone, and tetracycline residue detection in animal products marketed for human consumption [15], but species-level consumption data from LMICs are largely unavailable [16]. Reports are inconsistent, challenging international comparison due to lack of measurement standardization. A more complete picture of consumption variability is imperative in identifying targeted action toward optimizing usage.

Despite calls for establishing usage thresholds and targets [5,7,17], in the absence of an implementable, globally standardized methodology for quantifying consumption, progress toward setting, measuring, and achieving targeted usage benchmarks will remain limited.

Other challenges frequently shared by LMICs (Box 2) are equally important in realizing progress toward optimizing usage, and addressing them will require a foundational evidence base on consumption.

**Box 2. Challenges experienced by LMICs in optimizing antimicrobial consumption in animal production sectors**

- Low rates of AMR awareness and risk perception among farmers and veterinarians [15],
- Inconsistent policies governing antimicrobial use in animal production,
- Absent AMU regulation and enforcement structures enabling wide accessibility to critically important antimicrobials without prescription,
- Where a legal veterinarian–client–patient relationship exists governing prescription use of antimicrobials in animals, the link between prescription and sales presents a sales volume profit incentive, and
- Lack of systematic post-market quality surveillance that would enable recall of substandard and counterfeit veterinary antimicrobial products impacting therapeutic efficacy.

## Standardizing measurement methodologies in the context of LMICs

Multiple methodologies for quantifying usage have been variously employed, hindering data comparability across countries and production sectors. The OIE terrestrial animal health code, the guiding framework for its 180 member nations, outlines the minimum standard as measuring gross usage by weight of active ingredient per year [18].

Through the European Surveillance of Veterinary Antimicrobial Consumption (ESVAC) activity, European Union member states voluntarily report antimicrobial use by food animal–producing species [19]. The ESVAC guidance aims to harmonize antimicrobial consumption data and enable comparison with AMR surveillance data.

Antimicrobial consumption in food animals can be crudely estimated by quantifying the total weight of the active ingredient adjusted for biomass eligible for treatment, usually expressed as mg/kg population correction unit (PCU), where PCU is defined as total live and slaughtered animal weight per year in a prescribed geographic area. While this measurement of total volume of active ingredient per biomass considers neither the concentration of administered product nor its pharmacokinetics in individual species, it is a readily calculated estimate of usage. ESVAC guidelines use the mg/PCU methodology and introduce both defined daily dose in animals (DDD_vet_) and defined course dose in animals (DCD_vet_) [20], serving two functions: comparability across a range of species, usage, pharmaceutical formulations, and dosing regimens and aligning AMU methodologies in animals to those in humans, namely the defined daily dose (DDD) per 1,000 population per day endorsed as a global standard by WHO [21]. However, DDD_vet_ and DCD_vet_—proprietary ESVAC reporting nomenclature similar to animal defined daily dose (ADDD) and animal defined course dose (ADCD), respectively—are not suited to contexts in which measurement must also capture continuous-use, subtherapeutic dosing, as in LMICs where policies governing AGP use vary significantly.

## A phased capacity framework: Toward a global architecture for quantifying AMU in animal production

Accurate accounting of antimicrobial consumption in food-producing animals of LMICs must capture both therapeutic and nontherapeutic use, including AGPs and mass administration of antimicrobials delivered in medicated, premixed feed. Antimicrobial consumption monitoring is particularly important in aquaculture, in which mass administration of antimicrobials in medicated feed for disease prevention and control is a standard practice [22].

We propose a standardized, internationally-endorsed, phased approach that accounts for the context and currently available structures for collecting AMU data from food-producing animals—both terrestrial and aquatic species—in LMICs. The foundation is built upon the annual measurement of total sales of antimicrobials by class in mg/PCU, which can be derived from the total sales by class for member states reporting to the OIE divided by animal biomass data. Consistency in deriving the numerator—active pharmaceutical ingredient (API)—across multiple formulations of veterinary antimicrobials necessitates that products be managed through a standardized classification scheme, such as the Anatomical Therapeutic Chemical (ATCvet) system established for veterinary medical products [23]. Similarly, the animal biomass PCU denominator must employ a clearly and consistently applied calculation, using standardized production cycle and weight parameters [19] and accounting for animals locally produced, minus import and plus export of animals across borders. Leveraging existing, aggregate antimicrobial importation data; API domestic manufacturing and export data; antimicrobial sales data; and all-species animal biomass estimates publicly available [24] permit the rapid adoption of this foundational measurement scheme and bypasses substantial challenges in some LMICs of bottom-up quantification at the farm level, where legal requirements for identifying antimicrobials premixed into animal feed do not presently exist.

As countries establish refined data collection structures (Box 3), measurements could correspondingly evolve in subsequent phases to encompass more detailed parameters, including disaggregation by species and production type, and enhanced methodologies, such as number of animal defined daily doses (Table 1). Advanced phases could also involve collection sources more proximal to the site of usage—ultimately, at the individual animal or herd/flock/pond level—providing increasingly rich data sources directing optimal usage profiles and enabling usage audits and gap analysis for continued professional education. Achieving this detail requires both verifiable on-farm record keeping and longitudinal or serial, cross-sectional surveys capturing usage. Linkages with legislation and enforcement of appropriate incentive–penalty structures and establishment of an independent audit system will enhance compliance.

**Box 3. Application of the phased antimicrobial consumption monitoring approach in Thailand**

In 2015, Thailand’s International Health Policy Program and One Health partners established a human and animal national Surveillance for Antimicrobial Consumption (Thai SAC) system [25,26]. Thailand’s 1967 Drug Act mandates pharmaceutical importers and producers to submit an annual report on total volume of importation and manufacture of all medicines, including antimicrobials, to the Thai Food and Drug Administration. This total national antimicrobial sales data forms the foundation of Thai SAC, although the current reporting format does not require disaggregation by animal species. A 4-year (2014–2017) retrospective consumption study, with expected completion by mid-2018, will report mg active ingredient per PCU totaled across all animal species. Illustrating the application of an incremental, phased approach, in 2017, electronic information systems are being developed to facilitate e-reporting total sales of antimicrobial classes by animal species [27].

**Table 1:** Phased capacity approach to establishing surveillance for antimicrobial consumption in food animal production context of LMICs.

Parameter	Phase One	Phase Two	Phase Three	Phase Four	Phase Five
Methodology	mg/PCU	mg/PCU	mg/PCU	mg/PCU and ADDD	mg/PCU, ADDD, and ADCD
Unit of Measurement	mg/PCU*	mg/PCU disaggregated by species	mg/PCU disaggregated by species and production type	mg/PCU and number of ADDDs**	mg/PCU and number of ADDDs and ADCDs***
Data Requirements	- Total annual API sales (imported and manufactured minus exported) by class - Total annual animal biomass (locally produced minus imported, plus exported)	- Total annual API sales (imported and manufactured minus exported) by class and animal species - Total annual animal biomass by species (locally produced minus imported, plus exported)	- Total annual API sales (imported and manufactured minus exported) by class, animal species, and production type - Total annual animal biomass by species and production type (locally produced minus imported, plus exported)	Phase three data plus: - On-farm consumption data by species/type (mg/kg/day), route, and intended use, derived from product formulation, concentration, and unit size - On-farm production figures	Phase four data plus: - On-farm consumption data by species/type (mg/kg/course), route, and intended use, derived from product formulation, concentration, and unit - On-farm production figures
Data Source	- National registry of API sales - Animal production census data	- National registry of API sales - Distributor and end-user checks - Animal production census data	- National registry of API sales - Distributor and end-user checks - Animal production census data	Phase three sources plus: - On-farm usage reporting and production records, as collected from producers, veterinarians, pharmacies, feed mills	Phase three sources plus: - On-farm usage reporting and production records, as collected from producers, veterinarians, pharmacies, feed mills
Reporting Frequency	Annual	Annual	Annual	Annual	Annual

*PCU is defined as total live and slaughtered animal biomass in kg for a prescribed geographic area.

**Number of ADDDs is expressed in number/1,000 animals/year, where a standardized ADDD is the mean of all observed doses of antimicrobial across indications for a given species in mg/kg per day; presently, no global standardized ADDD exists and would need to be established for priority food-producing animal species.

***Number of ADCDs is expressed in number/1,000 animals/year, where a standardized ADCD is in mg/kg/treatment course.

Abbreviations: ADCD, animal defined course dose; ADDD, animal defined daily dose; API, active pharmaceutical ingredient; LMIC, low- and middle-income country; mg, milligram; PCU, population correction unit.

Technical assistance could be housed within existing or newly established coordinating bodies and should prioritize intraregional LMIC exchanges and solutions relevant to unique regional capacities and contexts. An intergovernmental panel on AMR has been suggested [28], similar to structures designed to catalyze global action on climate change. The United Nations ad hoc Interagency Coordination Group on Antimicrobial Resistance (IACG), formed at the 71st UN General Assembly in 2016, comprises agencies including the WHO-FAO-OIE tripartite, which can jointly coordinate technical and operational assistance between member countries [29].

## The path forward in optimizing AMU in food animal production sectors

Documenting usage—particularly for WHO-classified highest priority critically important antimicrobials—permits trends monitoring and identification of animal production sectors in which targeted interventions hold promise of arresting drivers of resistance. Conceivably, a reversion to susceptibility is achievable, as noted in *Salmonella enterica* Serovar Heidelberg following elimination of third-generation cephalosporins from poultry hatcheries in Quebec, Canada [30].

Establishing antimicrobial consumption monitoring systems in animal production sectors should be a central, near-term goal of multisectoral national AMR action plans aligned with the WHO Global Action Plan and the focus of advocacy and support from the UN FAO, OIE, and WHO tripartite. Embedding this approach within national AMR action plans promotes monitoring capacities endorsed across ministries, including those vested with budget authority, and costed, resourced, and prioritized for implementation.

In parallel, the WHO Joint External Evaluation (JEE) tool, which explicitly considers AMR and is increasingly being utilized by countries as an independent assessment of International Health Regulations (2005) capacity requirements [31], could develop an indicator evaluating AMU monitoring capacities. The JEE’s 1 (no capacity) to 5 (sustainable capacity) scoring structure is well matched to the phased, incrementally enhanced monitoring pathway presented here (Table 1), and incorporating a distinct indicator would ensure dedicated consideration of usage monitoring in human and animal sectors.

Antimicrobial consumption monitoring systems alone, however, will be insufficient to realize progress in directing prudent AMU in food-producing animals. Concurrently, support for strengthened farm and market chain biosecurity, enhanced livestock vaccination coverage, and improved uptake of good animal husbandry and nutrition practices will be necessary in achieving optimized usage, particularly in transitioning food animal production contexts of LMICs. New production facilities in LMICs should be designed to achieve high standards of husbandry and biosecurity, enabling more rapid phaseout of AGPs. Eliminating AGPs can be achieved at negligible cost to productivity, particularly in the context of such strengthened production systems [32].

The framework presented here should provide LMIC policy makers with high-quality antimicrobial consumption data that can be used to establish usage targets, while building incrementally enhanced monitoring capacities. The analyses derived from this approach could steer targeted policies optimizing antimicrobial consumption and scale back selective pressures currently driving AMR in animal production, with benefits expected to extend broadly across animal and human health.

## Disclaimer

The authors’ views expressed in this publication do not necessarily reflect the views of the United States Agency for International Development or the United States Government.

## Abbreviations

AGP: antimicrobial growth promoter
AMR: antimicrobial resistance
AMU: antimicrobial usage
API: active pharmaceutical ingredient
ATCvet: Anatomical Therapeutic Chemical classification for veterinary medical products
BRICS: Brazil, Russia, India, China, and South Africa
DCD_vet_: defined course dose in animals
DDD: defined daily dose
DDD_vet_: defined daily dose in animals
ESVAC: European Surveillance of Veterinary Antimicrobial Consumption
FAO: Food and Agriculture Organization of the United Nations
IACG: Interagency Coordination Group on Antimicrobial Resistance
JEE: Joint External Evaluation
LMIC: low- and middle-income country
OIE: World Organization for Animal Health
PCU: population correction unit
Thai SAC: Thailand’s Surveillance for Antimicrobial Consumption
